# Papillary carcinoma of the duodenum combined with right renal carcinoma: a case report

**DOI:** 10.1186/1477-7819-11-30

**Published:** 2013-02-01

**Authors:** Xuan Zhang, Zhen-hong Zhou, Shou-wang Cai, Jia-hong Dong

**Affiliations:** 1 Hospital and Institute of Hepatobiliary Surgery, Chinese PLA General Hospital, Chinese PLA Postgraduate Medical School, 28 Fu Xing Road, Beijing, 100853, People’s Republic of China; 2 Department of pathology, Chinese PLA General Hospital, Chinese PLA Postgraduate Medical School, 28 Fu Xing Road, Beijing, 100853, China

**Keywords:** Duodenum, Jejunal fistulization, Papillary carcinoma, Renal carcinoma, Renal resection

## Abstract

We report a case of papillary carcinoma of the duodenum combined with right renal carcinoma. A 58-year-old man underwent a physical examination that revealed intrahepatic and extrahepatic bile duct dilatation on B ultrasound. Intrahepatic bile duct dilatation could be seen on magnetic resonance imaging (MRI), but the head of the pancreas and distal bile duct showed no tumor signals, which led to a diagnosis of periampullary carcinoma and right renal carcinoma. Considering the trauma of pancreaticoduodenectomy combined with renal resection operation is greater, we carried out the laparoscopic right renal radical resection first, and then a pylorus-preserving pancreaticoduodenectomy was performed. However, postoperative intra-abdominal infections and bleeding occurred; our patient improved after vascular interventional microcoil embolization for the treatment of hemostasis. The second operation for celiac necrotic tissue elimination, jejunal fistulization and peritoneal lavage and drainage was performed 14 days latter. Our patient improved gradually and was discharged on the 58th postoperative day. There has been no tumor recurrence after a follow-up of 26 months.

## Background

Duodenal papilla carcinoma is classified as a periampullary carcinoma. Its early diagnosis is difficult because of the lesion site. It easily leads to bile duct obstruction because the cancer is in the ampulla; therefore the main clinical feature is progressive deepening painless jaundice [1-3]. There have been no previous reports of cases of duodenal papilla carcinoma combined with right renal carcinoma. Here, we describe such a case. Our patient was found to have intrahepatic and extrahepatic bile duct dilatation on B ultrasound without other symptoms initially, and was then hospitalized for further examination and treatment.

## Case presentation

**Figure 1:**
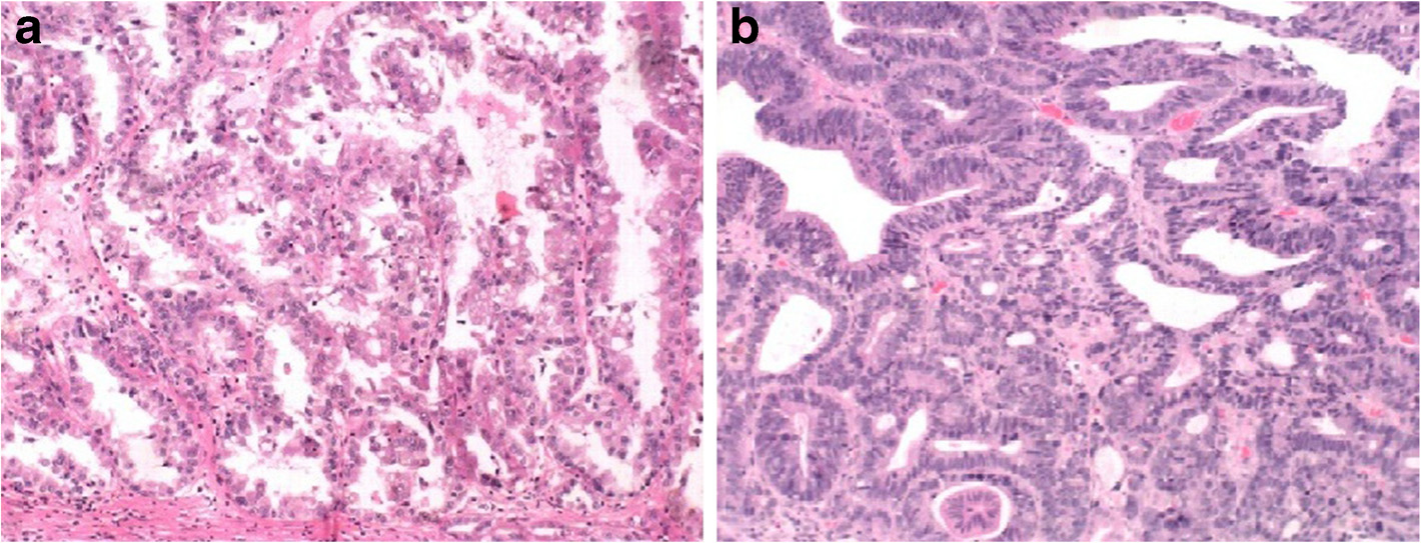
Pathological examination results: (a) right renal carcinoma, (b) papillary carcinoma of the duodenum.

A 58-year-old man was found to have intrahepatic and extrahepatic bile duct dilatation on B ultrasound on 12 June 2009, and was hospitalized for further examination and treatment on 20 June 2009. Our patient was 171 cm tall and weighed 86 kg. There was no anemia or jaundice in the palpebral or bulbar conjunctivas. The superficial lymph nodes were not palpable. The abdomen was flat without any palpable mass. He had had diabetes and hypertension that had been under regular medical control for the past 20 years. Abdominal ultrasonography was carried out and a 4.3 × 5.2 cm protruding tumor was found in the lower pole of the right kidney. The laboratory test results showed total bilirubin was 20.6 μmol/l and direct bilirubin was 10.3 μmol/l, the results of a magnetic resonance imaging (MRI) scan showed (1) a lesion in the lower pole of the right kidney, (2) cysts in the left renal area and (3) abnormal signals in the ampulla of Vater. Intrahepatic bile duct dilatation could be seen on the MRI scan, but the head of the pancreas and distal bile duct showed no tumor signals leading to a diagnosis of periampullary carcinoma and right renal carcinoma. Considering the trauma of pancreaticoduodenectomy combined with renal resection operation is greater, and laparoscopic resection of renal tumors is feasible with fast recovery, after weighing the risks of endoscopic retrograde cholangiopancreatography (ERCP) we carried out the laparoscopic right renal radical resection first without carrying out ERCP. After general anesthesia, our patient was operated on via a 2 cm skin incision under the costal margin on the right posterior axillary line; a retroperitoneal CO_2_ artificial pneumoperitoneum was established, the anatomical landmarks were observed under endoscope, the right renal artery was dissected, clipped with a blood vessel clip and cut. Then, the renal vein was dissected and cut, the adrenal gland was retained and the ureter was cut; the right kidney was then resected. Pathological examination results were as shown in Figure 1a. The clinical stage and TNM staging of the renal tumor was T1N0M0 and Robson stage I. Our patient recovered well and was discharged on the ninth postoperative day. Then, our patient presented with abdominal pain, fever, and body weight loss of about 10 kg the following month. He was hospitalized for further examination and treatment on 31 July 2009.

**Figure 2:**
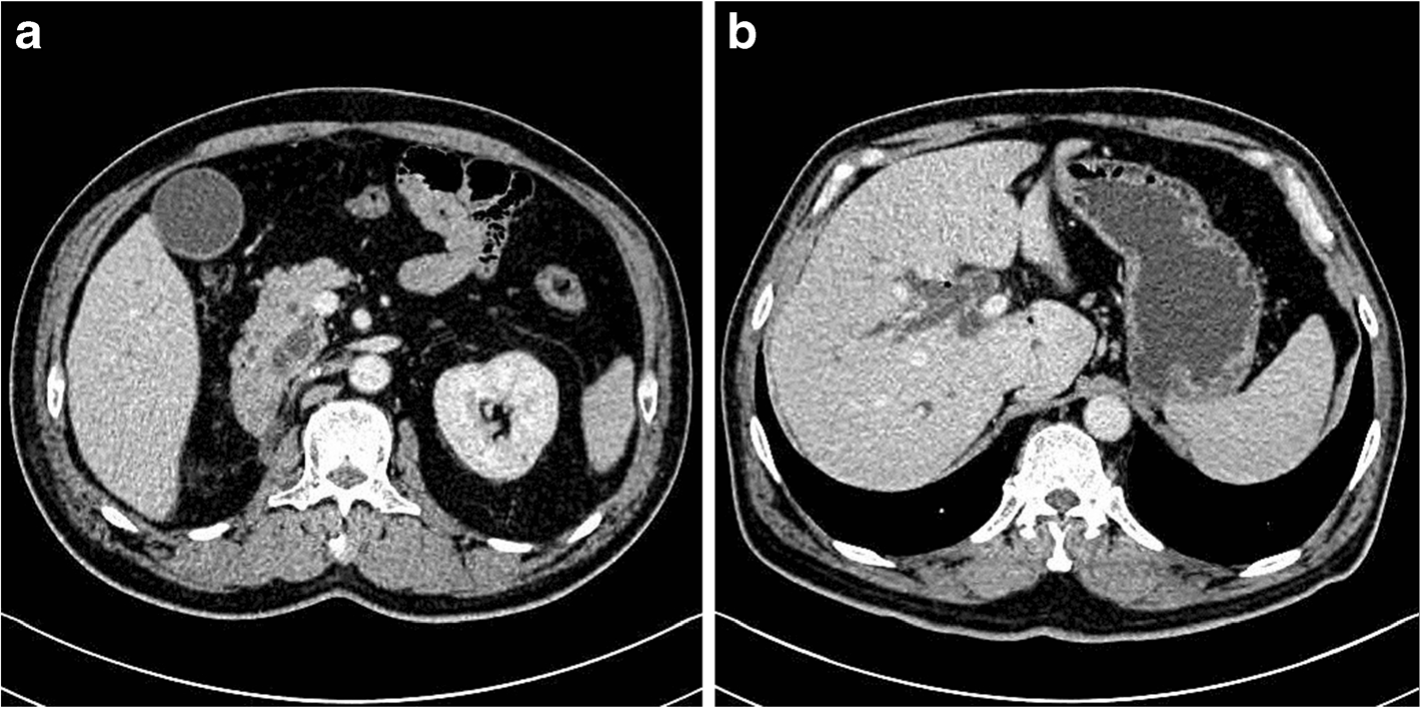
Computed tomography (CT) scan results showing (a) obstruction of the lower biliary tract and right kidney deficiency and (b) the intrahepatic bile duct dilatation.

**Figure 3:**
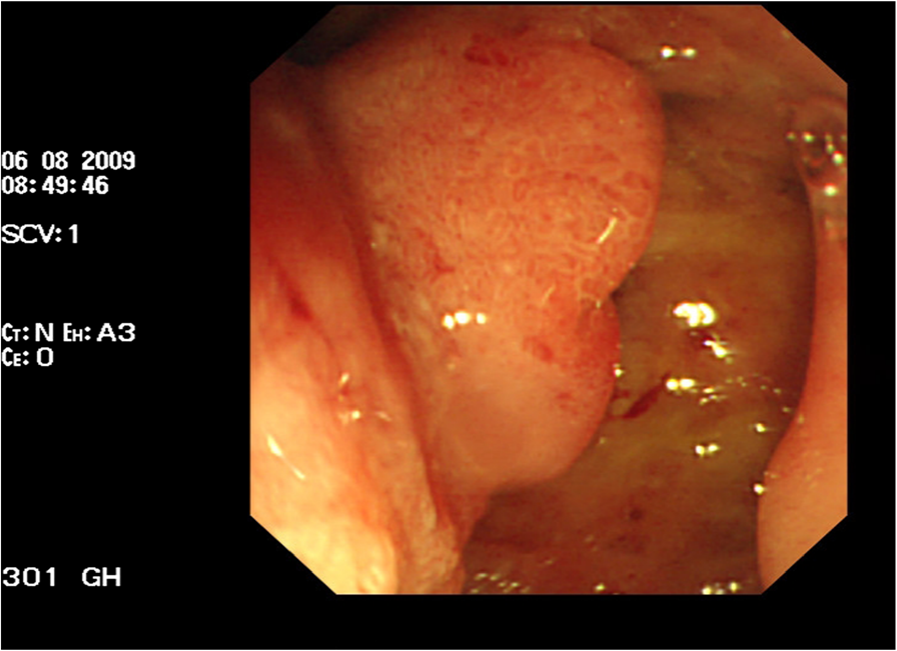
Endoscopic views of the duodenum.

ERCP showed that an infiltrative ulcerative mass was visible in the duodenal descending part and papilla involving the lumen half cycle; the mucosa was ulcerative and the intestinal wall was stiff. No nipple and wrinkled wall structure could be seen. We tried to carry out nipple angiography but were not successful. Papillary carcinoma of the duodenum was diagnosed (Figure 1b). Our patient presented fever and other symptoms of cholangitis at this time. Percutaneous transhepatic cholangiography drainage was carried out under the guidance of computed tomography (CT) (Figure 2a,b). Endoscopic views of the duodenum are shown in Figure 3. Postoperative anti-infection treatment (perazone sodium and sulbactam sodium) was applied. Our patient recovered well, then 30 days later the pylorus-preserving pancreaticoduodenectomy was carried out; the type was determined to be pancreatic duct jejunum anastomosis. However, postoperative intra-abdominal infections occurred on the sixth day and bleeding occurred in the peripheral arteriolar branch around the anastomosis on the eighth day; our patient improved after vascular interventional microcoil embolization for the treatment of hemostasis. The second operation was performed 14 days latter. A large number of dark red blood clots were seen on abdominal cavity exploration, mainly concentrated in the liver, gastric and pancreatic body and tail. Celiac necrotic tissues and blood clots were eliminated. At the same time, the gastrointestinal anastomosis had an approximately 1 cm split and bile was leaking from the biliary intestinal anastomosis, so jejunal fistulization was performed and peritoneal lavage and drainage was carried out repeatedly. The clinical stage and TNM staging of the pancreatic tumor was stage 1B and T2N0M0. Our patient improved gradually without undergoing chemotherapy and was discharged on the 58th postoperative day. There has been no tumor recurrence after a follow-up of 26 months.

## Conclusion

Duodenal papilla carcinoma is a rare finding, and comprises of less than 1% of all digestive malignant tumors. However, it is the second most common periampullary malignancy. The first clinical symptoms are always jaundice [4-6]. However, in this particular case, our patient had no clinical symptoms and intrahepatic and extrahepatic bile duct dilatation was only revealed on physical examination. The diagnosis of periampullary carcinoma and right renal carcinoma was made when our patient was hospitalized for further examination and treatment. He had diabetes and hypertension, kept under regular medical control for the past 20 years. This is therefore a very rare case. Radical resection is the only curative operation method for duodenal papilla carcinoma [7-9]. The trauma and risk are greater if resection of the two tumors is performed at the same time, so we carried out the laparoscopic right renal radical resection first. Our patient recovered well. However, he presented with symptoms of cholangitis in the following month. The pylorus-preserving pancreaticoduodenectomy was performed after we cured the cholangitis, then postoperative intra-abdominal infections and bleeding occurred. This may have been caused by the preoperative cholangitis or our patient’s diabetes. We then took appropriate treatment measures. Our patient improved gradually and was discharged on the 58th postoperative day. There has been no tumor recurrence after a follow-up of 2 years and 2 months.

## Consent

Our patient gave his written informed consent for this case report to be published.

## Competing interests

The authors have no conflicts of interest to declare.

## Authors’ contributions

XZ analyzed the data, and wrote the paper. ZZ interpreted the results. SWC discussed analyses, interpretation, and presentation. JD associated data collection and their interpretation. All authors read and approved the manuscript.
